# Age and sex differences in emergency department visits of nursing home residents: a systematic review

**DOI:** 10.1186/s12877-018-0848-6

**Published:** 2018-07-03

**Authors:** Annika Brucksch, Falk Hoffmann, Katharina Allers

**Affiliations:** 1 Department 11 Human and Health Sciences, University Bremen, Bremen, Germany; 2 Department of Health Services Research, Carl von Ossietzky University Oldenburg, Oldenburg, Germany

**Keywords:** Nursing, Nursing home (NH), Nursing home resident (NHR), Emergency department (ED), ED visit, Age, Sex, Hospital admission

## Abstract

**Background:**

Nursing home residents (NHRs) are often transferred to emergency departments (EDs). A great proportion of ED visits is considered inappropriate. There is evidence that male NHRs are more often hospitalised, but this is less clear for ED visits. It is unclear, which influence age has on ED visits. We aimed to study the epidemiology of ED visits in NHRs focusing on age- and sex-specific differences.

**Methods:**

A systematic review was carried out based on articles found in MEDLINE (via PubMed), CINAHL and Scopus. Articles published on or before Aug 31, 2017 were eligible. Two reviewers independently identified articles for inclusion. The quality of studies was assessed by the Joanna Briggs Institute critical appraisal tool for prevalence studies.

**Results:**

Out of 1192 references, we found seven studies meeting our inclusion criteria. Six studies were conducted in the USA or Canada. Overall, 29–62% of NHRs had at least one ED visit over the course of 1 year. Most studies assessing the influence of sex found that male residents visited EDs more frequently. All but one of the five studies with multivariable analyses reported a statistically significant positive association (with odds or rate ratios of 1.05–1.38). All studies assessed the influence of age. There was no clear pattern with some studies showing no association between ED visits and age and other studies reporting decreasing ED visits with increasing age or increasing proportions followed by a decrease in the highest age group. Studies used 85+ or 86+ years as the highest age category. Hospital admission rate ranged from 36.4 to 48.7%. There was no study reporting stratified analyses by age and sex. Only one study reported main diagnoses leading to ED visits stratified by sex.

**Conclusion:**

Male NHRs visit EDs more often than females, but there is no evidence on reasons. The association with age is unclear. Any future study on acute care of NHRs should assess the influence of age and sex. These studies should include large sample sizes to provide a more differentiated age categorisation.

**Trial registration:**

PROSPERO CRD42017074845.

**Electronic supplementary material:**

The online version of this article (10.1186/s12877-018-0848-6) contains supplementary material, which is available to authorized users.

## Background

Older people use emergency department (ED) services more often than persons of younger age [1]. In times of demographic changes, the burden on ED systems may further increase. In 2014, just over 1.4 million residents were living in US nursing homes, corresponding to 2.6% of the over-65 population and 9.5% of the over-85 population [2]. Compared with community dwellers nursing home residents (NHRs) have higher utilisation rates of EDs [3]. However, a large proportion of these ED presentations is considered inappropriate [4, 5]. Furthermore, it is questionable if benefits outweigh potential risks as ED visits of NHRs often result in unintended consequences and adverse outcomes like greater cognitive and physical decline or hospital-acquired infections [6, 7]. Approximately 50% of NHRs visiting EDs are discharged back to the nursing home without being hospitalised [8, 9] and almost one fifth of presentations followed by ED discharge had no diagnostic testing at all [9].

Although NHRs are typically older than 65 years, they represent a wide range of age groups up to over 100 years and a large proportion is female with increasing tendency in older age groups [10, 11]. Patterns of chronic diseases differ between sexes and across the age span in this population [10, 12], but most studies present epidemiologic measures aggregated for both sexes and potential differences between age groups are often not further examined. In their systematic review published in 2011, Gruneir et al. compared ED use by older adults to younger age groups, but they did not report on further age differences in NHRs [1]. Overall, the literature on age differences in hospitalisations of NHRs is inconclusive [13]. This seems also to be the case for ED visits with studies showing different findings [14, 15]. On the other hand, previous research showed that male NHRs are more often hospitalised than female NHRs [13, 16, 17], which might also apply for ED visits [15, 18].

The aim of this systematic review is to estimate the incidence and prevalence of ED visits in NHRs, focusing on age-specific and sex-specific patterns. We also gathered information on age-specific and sex-specific differences in reasons for ED visits, revisits and hospital admissions.

## Methods

A protocol of this systematic review was registered with PROSPERO (http://www.crd.york.ac.uk/PROSPERO; No.: CRD42017074845).

A systematic literature search was carried out for articles published on or before Aug 31, 2017. In a first step electronic databases including MEDLINE (via PubMed), CINAHL and Scopus were searched combining an adapted version of the search strategy of Hoffmann and Allers for NHRs [13] and a filter to retrieve studies related to EDs from Kung and Campbell [19]. The search strategy can be found in the Additional file 1. In a second step the reference lists of all identified articles were scanned for additional studies. There was no limitation regarding the time period.

### Inclusion and exclusion criteria

Studies were included if they assessed all-cause ED visits among NHRs and presented age-specific or sex-specific analyses on incidence or prevalence of ED visits or included one of these variables in crude or multivariable regression models.

Prevalence is defined as the proportion of NHRs admitted to EDs at a given point in time. Numerator is the total number of NHRs admitted to EDs and denominator is the total number of NHRs.

Incidence is defined as the measure of ED visits within a specified period of time and is usually expressed as a rate (e.g. per 100 or 1000 resident days, resident years, nursing home bed days). Numerator is NHRs’ ED visits and denominator is either the total number of NHRs under risk within a specified period of time or the accumulated time NHRs are at risk.

ED is defined as a hospital facility that provides unscheduled outpatient services to patients whose conditions require immediate care because of injury or illness or urgent medical conditions, and is staffed 24 h a day [20].

The current review considered prevalence studies, prospective and retrospective cohort studies and (randomised) controlled trials (provided that data from the comparison group were reported) for inclusion. There were no language restrictions and all articles published in languages other than English were translated.

Studies were excluded if they were restricted to specific groups of NHRs (e.g. specific diagnoses, specific levels of care, only NHRs with previous ED visits) or specific ED visits (e.g. specific diagnoses, only ED visits leading to hospitalisation).

### Study selection and data extraction

After removing duplicates, all titles and abstracts were screened independently by two reviewers (AB and KA) against the predefined inclusion criteria. In a next step, the full texts of all potentially relevant articles were assessed by the same reviewers. Any disagreement was resolved by discussion or by involving a third reviewer (FH). Data extraction was performed by one reviewer (AB) and verified by a second (KA).

We performed a descriptive synthesis of the identified studies due to the heterogeneity of the included studies.

### Risk of bias/quality assessment

All included studies were assessed by two independent reviewers (AB and KA) for methodological validity using a version of the prevalence critical appraisal instrument from the Joanna Briggs Institute (JBI) [21]. Any disagreements that arose between the reviewers were resolved by discussion or by a third reviewer (FH). We considered every study that met the inclusion criteria independent of their quality.

## Results

### Search results

**Fig. 1:**
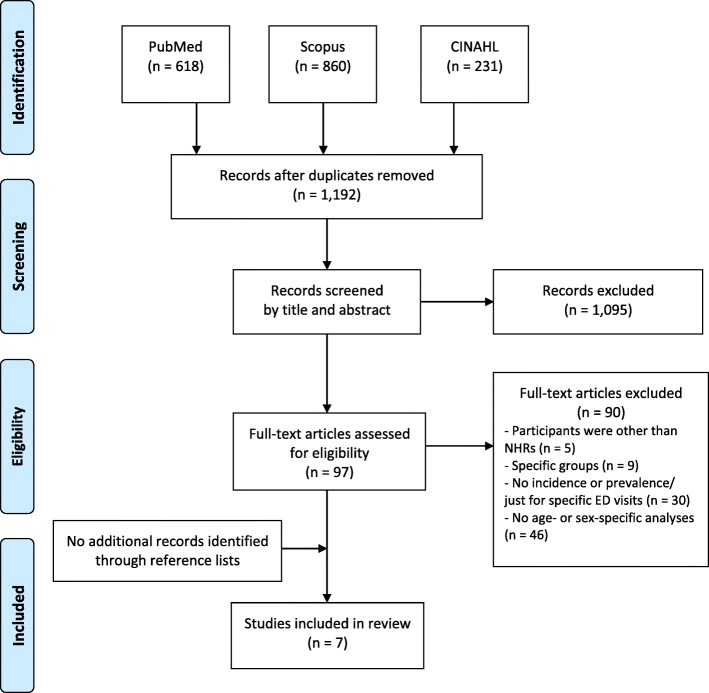
Flowchart of the literature search

After the removal of duplicates electronic searches identified a total of 1192 records. Screening for titles and abstracts resulted in the exclusion of 1095 records. Ninety-seven of the remaining potentially relevant articles were obtained in full text including four French, two Spanish, one Russian and one Hebrew article. Full text screening resulted in the exclusion of 90 articles and a total of seven articles were eligible for inclusion. No further articles were found in reference lists of the identified articles (Fig. 1).

### Study characteristics

The included studies were from the USA (*n* = 5) [15, 22–25], Sweden (*n* = 1) [14] and Canada (*n* = 1) [18]. The years of data used ranged from 1995 to 2009. Articles were published from 1998 to 2016. The studies included data from 719 to 132,753 NHRs. Follow-up periods ranged from 1 month to 3 years. Data on ED visits were most commonly obtained from administrative data or Minimum Data Set (*n* = 4) [22–25]. In the other three studies, data were collected by hospital staff [15, 18] or registered nurses [14]. The two articles from Stephens et al. reported findings from the same study but used different designs, analyses and number of participants [24, 25]. Therefore, both articles were included (Table 1).

**Table 1:** Characteristics of included studies

Author (year)	Country of origin	Study design (data source)	Year of data	Sample	Mean age of residents (% female)
Ackermann et al. (1998) [22]	USA	Retrospective chart review (ED records and data from the 1995 State of Georgia Annual Nursing Home Questionnaire)	1995	10 NHs with 1300 beds and 4 hospital-based EDs	65–74 years: 20.7%^a^ 75–84 years: 34.3%^a^ 85+ years: 29.0% (67.4%)
Hsiao and Hing (2014) [15]	USA	Cross-sectional study (data from the ED component of the 2001–2008 National Hospital Ambulatory Medical Care Survey (NHAMCS))	2001–2008	NHRs ≥ 65 years (no sample size given)	No data available
Kihlgren et al. (2014) [14]	Sweden	Cross-sectional follow-up study (RN’s documentation + Residents Assessment Instrument/Minimum Data Set (MDS))	2000–2002	719 NHRs ≥ 75 years from 24 NHs	Ø 85.8 years (71.0%)
LaMantia et al. (2016) [23]	USA	Retrospective cohort study (merged data set of Medicare and Medicaid claims and resident-level Minimum Data Set (MDS))	1999–2009	4491 long-stay NHRs ≥65 years	Ø 79.6 years (66.2%)^a^
McGregor et al. (2014) [18]	Canada	Retrospective cohort study (secondary administrative data on NHRs and ED records)	2005–2008	13,140 NHRs from 48 publicly funded NHs	Ø 83.1 years (66.6%)
Stephens et al. (2012) [24]	USA	Cross-sectional study (NH resident assessment data/Minimum Data Set (MDS) and Centers for Medicare and Medicaid Services (CMS) administrative claims)^b^	2006	132,753 NHRs ≥ 65 years from 2006 national random sample	65–75 years: 19.5% 76–85 years: 41.2% 86+ years: 39.3% (68.7%)^a^
Stephens et al. (2014) [25]	USA	Retrospective cohort study (Medicare administrative claims and NH resident assessment data/Minimum Data Set (MDS))^b^	2006	112,421 NHRs ≥ 65 years from 2006 national sample	65–75 years: 19.6% 76–85 years: 41.0% 86+ years: 39.4% (68.9%)

NH, nursing home; Ø, mean; ED, emergency department; RN, registered nurses

The Minimum Data Set (MDS) is part of the federally mandated process for clinical assessment of all residents in Medicare and Medicaid certified nursing homes

^a^Calculated from data given in the publication

^b^These articles used the same data set

### Quality appraisal of included studies

The quality assessment of all included studies and quality criteria are given in Table 2. The percentage of quality criteria answered with ‘Yes’ varied between 88 and 100%. The sample was representative of the target population in all studies. All study participants were recruited in an appropriate way and all studies used objective criteria assessing ED visits. All studies except one used appropriate statistical analyses. Overall, questions were answered predominantly with ‘Yes’, because almost all studies used administrative data. The remaining two studies including one survey mentioned the response, but gave no details regarding a sufficient coverage of the identified sample.

**Table 2:** Summary of quality assessment

Author (year)	1	2	3	4	5	6	7	8	9
Ackermann et al. (1998) [22]	Yes	Yes	Yes	Yes	Not applicable	Yes	Yes	No	Yes
Hsiao and Hing (2014) [15]	Yes	Yes	Yes	Yes	Not clear	Yes	Yes	Yes	Yes
Kihlgren (2014) [14]	Yes	Yes	Yes	Yes	Not clear	Yes	Yes	Yes	Yes
LaMantia et al. (2016) [23]	Yes	Yes	Yes	Yes	Not applicable	Yes	Yes	Yes	Yes
McGregor et al. (2014) [18]	Yes	Yes	Yes	Yes	Not applicable	Yes	Yes	Yes	Yes
Stephens et al. (2012) [24]	Yes	Yes	Yes	Yes	Not applicable	Yes	Yes	Yes	Yes
Stephens et al. (2014) [25]	Yes	Yes	Yes	Yes	Not applicable	Yes	Yes	Yes	Yes

Quality appraisal criteria [21]:

1. Was the sample frame appropriate to address the target population?

2. Were study participants sampled in an appropriate way?

3. Was the sample size adequate?

4. Were the study subjects and setting described in detail?

5. Was the data analysis conducted with sufficient coverage of the identified sample?

6. Were valid methods used for the identification of the condition?

7. Was the condition measured in a standard, reliable way for all participants?

8. Was there appropriate statistical analysis?

9. Was the response rate adequate, and if not, was the low response rate managed appropriately?

### Resident characteristics

Studies commonly included all residents (*n* = 2) [20, 24] or all residents aged 65 and older (*n* = 4) [15, 23–25]. One study included only residents aged 75 and older [14]. Two studies investigated residents with varying stages of cognitive impairment and dementia [23, 25]. The mean age of NHRs ranged from 79.6 to 85.8 years [14, 18, 23] and between 29.0–39.4% were aged 85 or 86 years and older [22, 24, 25]. In these six studies between 66.2 and 71.0% were females. In one study [15] baseline data on age and sex were not reported.

### Frequency of ED visits

All but one study [25] examined some measure of all-cause ED visits (Table 3). The proportion of NHRs admitted to the ED ranged from 29 to 62% over a one-year period [14, 23, 24]. The incidence of ED visits ranged between 62.6 and 215.5 per 100 resident years [14, 15, 18, 22, 23], with three of the studies ranging between 110 and 150 ED visits per 100 resident years [14, 15, 22]. One study found a trend towards increasing ED visits over time with a rate of 146.0 ED visits per 100 resident years in 2001 compared to 215.5 ED visits per 100 resident years in 2008 [23].

**Table 3:** ED visits of NHRs

Author (year)	Prevalence, incidence or number of ED visits and follow-up	Age-specific and sex-specific analyses		Admission to hospital	Number of ED visits per resident
		Prevalence or incidence	Regression/model		
Ackermann et al. (1998) [22]	110 visits per 100 resident years (1488 ED visits made by 873 residents) Follow-up: 1 year		Holm’s Sequential Rejection Algorithm Men had a 17% higher overall ED use rate than women (*p* = 0.025). No association between the overall ED use rate of a NH and the age distribution of its patients.^a^	Admitted to hospital: 42.4% Not admitted: 56.3% Died: 1.3% Admitted to hospital: Age ≤ 64 years: 36.6% 65–74 years: 44.9% 75–84 years: 41.8% 85+ years: 44.6% Sex Female: 41.6% Male: 43.9%	Number of ED visits per resident 1: 60.5% 2: 22.3% 3: 9.5% 4: 4.2% 5+: 3.4%
Hsiao and Hing (2014) [15]	123.2 visits per 100 resident years (4970 ED visits) Follow-up: 1 month	Number of visits per 100 resident years Age 65–74 years: 153.2 (95% CI 138.1–168.3) 75–84 years: 124.3 (95% CI 115.5–133.1) 85+ years: 113.0 (95% CI 105.0–121.0) Sex Female: 111.6 (95% CI 104.5–118.7) Male: 154.5 (95% CI 142.3–166.7)		Admitted to hospital: 48.7% Not admitted: 33.5% Multivariable analysis Admitted to hospital: Age 65–74 years: Reference 75–84 years: OR = 0.95 (95% CI 0.75–1.20) 85+ years: OR = 1.02 (95% CI 0.82–1.27) Sex Male: OR = 1.10 (95% CI 0.93–1.30)	Proportion of residents seen in ED less than 72 h ago: 2.4% (95% CI 1.9–3.0)
Kihlgren et al. (2014) [14]	150 visits per 100 resident years NHRs with at least one ED visit: 29% (314 ED visits made by 209 residents) Follow-up: 1 year	Age 75–84 years: 25% 85+ years: 30% (*p* = 0.213) Sex Female: 28% Male: 33% (*p* = 0.196)			Number of ED visits per resident 1: 71% (number of referrals ranged between 1 and 7) Average number of referrals: Female: 1.4 Male: 1.7
LaMantia et al. (2016) [23]	2001: 146.0 visits per 100 resident years 2008: 215.5 visits per 100 resident years^b^ NHRs with at least one ED visit: 47% Follow-up: 1 year		Multivariable cox proportional hazard regression Age HR = 0.98 (95% CI 0.98–0.99; *p* < 0.0001) Sex Male: HR = 0.95 (95% CI 0.87–1.05; *p* = 0.3610)^c^	Admitted to hospital: 36.4% Not admitted: 63.1% Died: 0.52%	
McGregor et al. (2014) [18]	62.6 visits per 100 resident years^c,d^ (10,710 ED visits) Follow-up: 3 years		Poisson regression Adjusted (for sex and age) Age IRR = 1.00 (95% CI 1.00–1.00) Sex Male: IRR = 1.38 (95% CI 1.28–1.49)		
Stephens et al. (2012) [24]	NHRs with at least one ED visit: 62% (82,335 ED visits) Follow-up: 1 year	Age 65–75 years: 62.7% 76–85 years: 63.9% 86+ years: 59.7% Sex Female: 60.5% Male: 65.3%	Multivariable logistic regression Age 65–75 years: Reference 76–85 years: OR = 1.11 (95% CI 1.07–1.15; *p* ≤ 0.0001) 86+ years: OR = 1.03 (95% CI 0.99–1.07) Sex Male: OR = 1.05 (95% CI 1.02–1.09; *p* ≤ 0.001)		
Stephens et al. (2014) [25]	ED visits: 112,119 Follow-up: 1 year		Poisson regression Adjusted Age 65–75 years: Reference 76–85 years: IRR = 0.95 (95% CI 0.93–0.97; *p* < 0.0001) 86+ years: IRR = 0.90 (95% CI 0.88–0.92 *p* < 0.0001) Sex Male: IRR = 1.14 (95% CI 1.12–1.17; *p* < 0.0001)	Admitted to hospital: 44.16% Multivariable logistic regression Admitted to hospital: Age 65–74 years: Reference 76–85 years: OR = 1.07 (95% CI 1.02–1.13) 86+ years: OR = 1.11 (95% CI 1.05–1.17) Sex Male: 1.10 (95% CI 1.06–1.15)	

IRR, Incidence Rate Ratio; OR, Odds Ratio; HR, Hazard Ratio

^a^No other information available

^b^Authors reported per 1000 NH bed days

^c^Calculated from data given in the publication

^d^For-profit facilities: 69/100 resident years, 1.9/1000 resident days; non-profit facilities: 70/100 resident years, 1.93/1000 resident days; public owned facilities: 51/100 resident years, 1.39/1000 resident days

All studies assessed the influence of sex. Two studies stratified their results for males and females [14, 15] and four studies conducted regression analyses including sex in the model [18, 22, 23, 25]. One study reported both [24]. Most studies came to the conclusion that male NHRs visit EDs more often than females. One study reported that 65.3% of the male NHRs visited the ED over a one-year period compared to 60.5% of the female NHRs [24], while another study reported a prevalence of 33 and 28% for male and female NHRs [14], respectively. A further study found an incidence of 154.5 per 100 resident years for male and 111.6 per 100 resident years for female NHRs [15]. All but one of the multivariable analyses reported a statistically significant positive association between male sex and ED visits (odds or rate ratio: 1.05–1.38) [18, 22, 24, 25]. The other study analysed factors predicting time to first ED visit in the year after study entry and found no association between male sex and ED visits [23].

All seven studies assessed the influence of age. Three of the studies stratified their results by age [14, 15, 24]. One study reported decreasing incidences of ED visits with rising age (65–74 years: 153.2, 75–84 years: 124.3, 85+ years: 113.0 ED visits per 100 resident years) [17]. Another study found a higher prevalence of ED visits (30%) in the age of 85 years and older compared to the age of 75–84 years (25%), but this finding was not statistically significant [14]. The third study underlined a slightly increasing prevalence of ED visits from 65-75 years (62.7%) to 76–85 years (63.9%) of age and a slightly decreasing proportion in those aged 86 years and older (59.7%) [24]. Five studies included age in regression analyses [18, 22–25]. Whereas Stephens et al. found significantly lower odds of any ED visit for the age of 65–75 years compared to the age of 76–85 years, this was not statistically significant for the age group of 85 years and older [24]. One study found that higher age was associated with lower rates of total ED visits [25], while two other studies did not show any association between age and overall ED use rate [18, 22]. One study found that age influences the time to first ED visit [23]. However, of the five studies that conducted multivariable analyses, only two used the same age categories (65–75, 76–85 and 86+ years) [24, 25], two incorporated age as a continuous variable [18, 23], and the last did not clearly report how age was included in the model, but probably also continuously [22].

### Reasons for ED visits

Of the seven included studies, one reported diagnoses for ambulatory care sensitive conditions (ACSC), which made up 14.6% of all ED transfers. The two most common diagnoses were kidney/urinary tract infection (4.9%) and congestive heart failure (3.2%) [15]. Two further studies gave reasons for all ED transfers [14, 22]. Most common reasons were respiratory symptoms (14.4%), altered mental status (10.1%), gastrointestinal symptoms (9.9%) and falls (8.2%) in the study of Ackermann et al. [22]. Kihlgren et al. reported falls (22.6%), cardiovascular and cerebrovascular problems (16.2%), gastrointestinal symptoms (11.8%), fever and infections (11.1%) as the most common reasons [14]. However, with 20.7% the number of missings on reasons for referrals was high.

One of these two studies reported reasons leading to ED visits stratified by sex [14]. For women, falls were the most frequent reason (25.4%) followed by cardiovascular and cerebrovascular problems (15.4%) and for men falls as well as cardiovascular and cerebrovascular problems (17.7% each) were most common. No study stratified reasons for ED visits by age or ACSC diagnoses by sex or age.

One study reported that 23.8% of NHRs (24.7% of male and 23.4% of female NHRs) had at least one potentially preventable ED visit [24]. The authors also reported slightly decreasing proportions having at least one avoidable ED visit with increasing age (65–75 years: 26.0%, 76–85 years: 24.7% and 86+ years: 21.7%).

### Revisits

Another three studies showed the following pattern of ED revisits. One study reported that 60.5% had one ED visit, 22.3% had two visits and 17.2% had three or more visits over the course of 1 year [22]. The second study found that 2.4% of the study population had been seen in ED less than 72 h ago, while 87.3% were not seen again (for 10.3% the status was unknown) [15]. Only one study stratified the results by sex, showing that female NHRs had 1.4 revisits and male NHRs 1.7 revisits during the one-year study period [14]. There was no study that stratified revisits by age (Table 3).

### Hospital admission

Four studies reported subsequent hospital admissions of NHRs following ED visits. The proportion of hospitalisation ranged from 36.4 to 48.7% [15, 22, 23, 25] and between 0.5 and 1.3% of NHRs died in the ED [22, 23]. Three studies [15, 22, 25] reported on differences of age and sex. While two studies found that patients admitted to hospital did not vary by age and sex [15, 22], one other study reported that male sex and advanced age were associated with higher odds of hospitalisation [25] (Table 3).

## Discussion

### Summary of main findings

This systematic review analysed age-related and sex-related ED presentations in NHRs and found only very few studies assessing these patterns. Most studies examining sex differences in ED visits found that male NHRs visited EDs more often than females. The influence of age was less clear with some studies showing no association and others reporting decreasing ED visits with increasing age or increasing proportions followed by a decrease in the highest age group. However, comparability is limited as some of the included studies used age as a continuous variable. There was no study which reported stratified analyses by age and sex.

### Comparison with the existing literature

We found a wide range between 29 and 62% of NHRs that had at least one ED presentation over a one-year period and the proportion of NHRs being admitted to hospital ranged from 36.4 to 48.7%. These findings and the variability are comparable with the literature and might also reflect facility-level variations [1, 4, 6, 9]. Furthermore, the existing literature is also heterogeneous with respect to methods, time periods and populations. This is important to keep in mind, when comparing and interpreting findings between different studies.

Like in our recent review on hospitalisations of NHRs [13], we also found that male NHRs visited EDs more often than females in this systematic review. We only included studies assessing all NHRs in the denominator instead of only ED patients, because the latter might have led to the conclusion that women visit EDs more frequently [9, 26]. However, this is explained by the fact that a large proportion of NHRs is female. Although not all included studies found statistically significant effects, which might also be due to small sample sizes, a clear trend was seen. The strongest influence of male sex with a rate ratio of 1.38 was shown by McGregor et al. [18], but this result was not further discussed by the authors as these differences were not the focus of their study. This was also the case in the other included studies. In their review on trends and appropriateness of ED use by older adults, Gruneir et al. [1] did not even mention sex as a potential factor. This was also the case in a more recent review by Trahan et al. on factors influencing decision-making on transitions of NHRs to EDs [27]. Although the authors identified residents and family factors as one of five categories, no sociodemographic factors were considered. Because hospital as well as ED use is higher for males, decisions to transfer seem to be made in the nursing home. Only one of the included studies reported reasons leading to ED visits stratified by sex and found that falls were more often the reason to transfer female NHRs (25.4% vs. 17.7%) with men having slightly higher proportions in several other categories [14]. But for one fifth of transfers no reason for referral was available. The proportion of potentially avoidable ED visits was high and ranged between 25 and 55% [6, 28, 29]. One of our included studies stratified the proportion of NHRs having at least one potentially avoidable ED visit by sex and found only marginal differences between males and females [24]. Furthermore, facility-level variation across nursing homes has been shown to influence health care including ED transfers [4, 30], but it is unclear whether sex differences also depend on facility-levels. Future studies should assess which ED transfers vary between sexes.

On the other hand, the influence of age on ED visits was inconsistent in the included studies. There is some evidence of a decreasing influence of age above about 85 years, but this was not shown or assessed in all studies. Such heterogeneous findings were also found in the literature on ED use of elderly patients irrespective of nursing home stay [20, 26, 31, 32]. In our systematic review on hospitalisations of NHRs we also concluded that the influence of age was inconclusive due to methodological differences [13]. In a large cohort of German NHRs, we recently found that hospitalisation rates declined with increasing age even up to 95+ years, but this effect was much more pronounced before nursing home entry [33]. These inconsistent findings on the influence of age in the literature may be on the one hand due to different outcomes. Two of the studies included in our review assessed prevalences [14, 24] and one assessed incidences [15] of ED visits. The included studies also used different statistical analyses (e.g., logistic, poisson or cox proportional hazard regression). On the other hand, age was mostly assessed as a continuous variable in regression models, although no linear effect might exist, or with only few categories. Three out of seven studies included in this review conducted multivariable analyses including age as a continuous variable [18, 22, 23] and the other four studies used 85+ or 86+ years as the highest age category [14, 15, 24, 25]. As NHRs typically represent a much wider age span ranging between under 65 up to over 100 years [10, 11], more differentiated age-specific patterns have to be assessed. When further taking into account that women have longer life expectancies than men resulting in a higher percentage of women at older ages [10], both sociodemographic variables have to be considered simultaneously. This is important because the individual effects of age and sex cannot be determined otherwise and confounding or effect modification is possible. However, none of the included studies stratified their results on ED visits by age and sex.

In our recent systematic review on age and sex differences in hospitalisations of NHR, we encouraged further research on the influence of sociodemographic characteristics on ED visits of NHRs [13]. As ED visits are frequent events in NHRs and only about half of the visits result in hospital admission [1, 9, 23], acute care in EDs plays an important role. Interestingly, we only found seven studies (of which the two articles from Stephens et al. even reported findings from the same study [24, 25]) on age or sex differences in ED visits of NHRs as compared to 20 in our review on hospitalisations [13]. Moore et al. already pointed out in 2012 [10], that understanding age and sex dependent patterns in NHRs is the key to optimize individual care. Therefore, we strongly encourage that any further research on health care of NHRs should include large sample sizes and consider differences between these sociodemographic characteristics. Only after exploring reasons for age and sex specific patterns of ED visits, conclusions for health administrators and clinicians can be drawn.

### Strengths and limitations

We conducted the first systematic review examining age and sex differences in the epidemiology of ED visits of NHRs using a comprehensive search strategy. We did not restrict our search to specific languages. Furthermore, we screened reference lists of all included articles. Nevertheless, there is still the possibility that we could have missed studies that comprised information about ED visits of NHRs by sex or age. However, we screened the full text of about 100 articles that might have reported such information but finally included only seven relevant studies in our systematic review. The interpretation of our findings is limited by the inclusion of very heterogeneous studies in terms of populations, time frames and estimates (e.g. crude or standardised frequencies and multivariable regression models) which might have accounted for some of the differences in the results. The studies included are also too few to assess time trends or differences between countries.

Since there are no established and validated tools for studies on prevalence and incidence, quality assessment was carried out by using the critical appraisal instrument of the JBI [21]. This tool rather gives an overview on the study characteristics than evaluating methodological quality and the application to studies using administrative data is difficult because they generally have, for example, an adequate response or an appropriate sample size. Further research on tools for quality assessment of studies examining prevalences or incidences is needed.

## Conclusion

Our knowledge on age and sex differences in acute care use of NHRs is still limited. We only found seven studies meeting our inclusion criteria. Male NHRs visit EDs more often than females, but reasons for that are not analysed or discussed in the corresponding studies. The influence of age is less clear, which might be due to very heterogeneous age categorisations. Taken together, any future studies on acute care of NHRs should assess the influence of sociodemographic characteristics like age and sex. These studies should include large sample sizes to provide a more differentiated age categorisation.

## Additional file

Additional file 1: Search strategy. (DOCX 15 kb)

## Abbreviations

ED: Emergency department
JBI: Joanna Briggs Institute
NH: Nursing home
NHR: Nursing home resident
OR: Odds Ratio
RN: Registered nurses
