# Supplementary material for: Age and sex differences in emergency department visits of nursing home residents: a systematic review
Source: BMC Geriatr. 2018 Jul 3;18:151. doi: 10.1186/s12877-018-0848-6 (PMC6029412; doi:10.1186/s12877-018-0848-6)
Supplement: Supplementary file 1 — Search strategy. (DOCX 15 kb) [file 12877_2018_848_MOESM1_ESM.docx]

**Additional file 1** Search strategy.

**PubMed**

(((nursing home [mesh]) AND (resident[tiab] or residents[tiab])) OR (("nursing home" [tiab] OR "nursing homes" [tiab] OR "nursing facility"[tiab] OR "nursing facilities" [tiab]) AND (resident[tiab] OR residents[tiab])) OR (("long‐term care facility"[tiab] OR "long‐term care facilities"[tiab]) AND (resident[tiab] OR residents[tiab])) OR "home for the aged"[tiab] OR "homes for the aged"[tiab] OR "institutional* care"[tiab]) AND (emergency treatment[mesh] OR emergency medicine[mesh] OR emergency health service[mesh] OR evidence based emergency medicine[mesh] OR emergency nursing[mesh] OR emergency care [mesh] OR emergency ward [mesh] OR emergency[mesh] OR (emergicent*[tiab] OR ((emergenc*[tiab] OR ED[tiab]) AND (room*[tiab] OR accident[tiab] OR ward[tiab] OR wards[tiab] OR unit[tiab] OR units[tiab] OR department*[tiab] OR physician*[tiab] OR doctor*[tiab] OR nurs*[tiab] OR treatment*[tiab] OR visit*[tiab])) OR (triage[tiab] OR critical care[tiab] OR (trauma[tiab] AND (centre*[tiab] OR center*[tiab] OR care[tiab])))))

**Scopus**

( ( TITLE-ABS-KEY ( "nursing home" OR "nursing facility" OR "long-term care facility" ) AND TITLE-ABS ( resident ) ) OR ( TITLE-ABS-KEY ( "home for the aged" ) ) ) AND ( ( TITLE-ABS-KEY ( "emergency treatment" OR "emergency medicine" OR "emergency health service" OR "evidence based emergency medicine" OR "emergency nursing" OR "emergency care" OR "emergency ward" OR "emergency" ) ) OR ( TITLE-ABSKEY ( emergicent* OR emergenc* OR ed ) ) OR ( TITLEABS ( ( emergency W/1 room* ) OR ( emergency W/1 accident ) OR ( emergenc y W/1 ward ) OR ( emergency W/1 wards ) OR ( emergency W/1 unit ) OR ( e mergency W/1 units ) OR ( emergency W/1 department* ) OR ( emergency W/1 physician* ) OR ( emergency W/1 doctor* ) OR ( emergency W/1 nurs* ) OR ( e mergency W/1 treatment* ) OR ( emergency W/1 visit* ) ) ) OR ( TITLE-ABSKEY ( triage OR "critical care" OR ( trauma W/1 centre* OR center* OR care ) ) ) )

**CINAHL**

((((MH "Nursing Homes+") AND (resident OR residents)) OR ((nursing N1 (home* OR facilit*)) AND (resident OR residents)) OR ("long‐ term care facilit*" AND (resident OR residents)) OR "home* for the aged" OR "institutional* care")) AND ((MH "Emergency treatment+" OR MH "emergency medicine+" OR MH "emergency health service+" OR MH "evidence based emergency medicine+" OR MH "emergency nursing+" OR MH "emergency care+" OR MH "emergency ward+" OR MH "emergency+ ") OR (emergicent* OR (emergenc* OR "ED") AND N1 (room* OR accident OR ward OR wards OR unit OR units OR department* OR physician* OR doctor* OR nurs* OR treatment* OR visit*) OR (triage OR critical care OR (trauma N1 (centre* OR center* OR care)))))
